# Absence or presence? Complexities in the donor narratives of single mothers using sperm donation

**DOI:** 10.1093/humrep/dev275

**Published:** 2015-11-05

**Authors:** S. Zadeh, T. Freeman, S. Golombok

**Affiliations:** Centre for Family Research, University ofCambridge, Free School Lane, Cambridge CB2 3RQ, UK

**Keywords:** Single motherhood, sperm donation, family narratives, information provision, identity-release donation

## Abstract

**STUDY QUESTION:**

How do single mothers who have conceived a child via anonymous or identity-release sperm donation represent the donor?

**SUMMARY ANSWER:**

While the majority of mothers described their anonymous and identity-release donors as symbolically significant to their families, others were more likely to emphasize that their lack of information limited their thoughts about him.

**WHAT IS KNOWN ALREADY:**

There is limited understanding of the factors that impact upon how single mothers represent the donor, and whether or not they are determined by specific donor programmes (anonymous or identity-release).

**STUDY DESIGN, SIZE, DURATION:**

Qualitative interviews were conducted with 46 women who had treatment at a UK licensed fertility clinic during the years 2003–2009. Twenty mothers (43%) had used an anonymous donor, and 26 (57%) had used an identity-release donor.

**PARTICIPANTS/MATERIALS, SETTING, METHODS:**

Among the 46 mothers interviewed, all had at least one child conceived via donor insemination who was between the ages of 4 and 9 years. Mothers were heterosexual and were currently without a live-in and/or long-term partner. Interview data were analysed qualitatively according to the principles of thematic analysis.

**MAIN RESULTS AND THE ROLE OF CHANCE:**

Findings indicated marked diversity in single mothers' representations of the donor. Most (*n* = 27) mothers talked about the donor as symbolically significant to family life and were likely to describe the donor as (i) a gift-giver, (ii) a gene-giver and (iii) a potential partner. Others (*n* = 16) talked about the donor as (i) unknown, (ii) part of a process and (iii) out of sight and out of mind. There were mothers with anonymous and identity-release donors in each group. Several mothers explained that their feelings about the donor had changed over time.

**LIMITATIONS, REASONS FOR CAUTION:**

All mothers conceived at a licensed fertility clinic in the UK. Findings are limited to individuals willing and able to take part in research on donor conception.

**WIDER IMPLICATIONS OF THE FINDINGS:**

The study offers greater insight into the factors influencing the donor narratives produced in single-mother families. It has implications for the counselling and treatment of single women seeking fertility treatment with donor gametes in both anonymous and identity-release programmes. Given that the number of clinics offering identity-release programmes worldwide seems to be increasing, the finding that single women may have varying preferences with regard to donor type, and varying interest levels with regard to donor information, is important. It is recommended that clinicians and other fertility clinic staff guard against making assumptions about such preferences and any thoughts and feelings about the donor or donor information on the basis of marital status.

**STUDY FUNDING/COMPETING INTEREST(S):**

This study was funded by the Wellcome Trust [097857/Z/11/Z]. The authors have no conflicts of interest to declare.

## Introduction

Donor insemination is a form of third-party assisted reproduction that enables individuals and couples to conceive using donor sperm. Although initially established in order to assist heterosexual couples to become parents, a significant proportion of those who now seek fertility treatment with donated gametes are women without a male partner (De Wert *et al.*, 2014; HFEA, 2014). Coupled with this demographic shift are widespread legislative changes to the type of donation that the users of donor insemination are able to receive. In addition to those cases in which couples or individuals use a donor who is known to them (such as a family member or friend), all donations of gametes in the UK, the Netherlands and the Australian state of Victoria are now such that a donor's identity is available for release once a child reaches the age of 18 years (Goldberg and Scheib, 2015). In the UK, the transition from anonymous to identity-release donation took place in 2005. The national regulator, the Human Fertilization and Embryology Authority (HFEA), now advises that clinics encourage donors to provide as much information as possible about themselves. It is further recommended that clinic staff alert donors to the importance of the information they provide to those born as a result of their donation (HFEA, 2013). Relatedly, the number of clinics offering identity-release donation in the USA seems to be increasing (Scheib and Cushing, 2007), with some States, such as Washington, recently introducing a law that requires donors to be identity-release unless they choose to ‘opt out’.

Studies measuring the preference rates for known, identity-release or anonymous donation have shown that heterosexual couples may be less likely than single women and lesbian couples to actively choose an identity-release donor, and less likely to attribute importance to identifying information about him (Scheib *et al.*, 2003; Brewaeys *et al.*, 2005; Godman *et al.*, 2006). One study found that single women rated information about donor age, occupation, interests and feelings about future contact as more important on average than women in heterosexual and lesbian couples (Rodino *et al.*, 2011b). Other research has shown that heterosexual couples may be especially likely to request information about the physical appearance of prospective donors (Bielawska-Batorowicz, 1994; Becker, 2000), and that both heterosexual and lesbian couples may seek to ‘match’ the physical characteristics of the donor to the non-biological parent in their family (Scheib *et al.*, 2000; Nordqvist, 2010; Braverman and Frith, 2014). This practice has also been widely described by recipients of donor insemination as encouraged by fertility clinic staff in both the USA and the UK (Ehrensaft, 2000; Chabot and Ames, 2004; Kirkman, 2004a; Becker *et al.*, 2005; Nordqvist, 2012).

In keeping with this literature, research of a qualitative nature has also indicated that heterosexual couples may minimize the role of the donor in their family narratives (Becker, 2000; Kirkman, 2003, 2004a, b; Grace and Daniels, 2007; Burr, 2009; Wyverkens *et al.*, 2015). It has moreover been suggested that single women may be more likely to emphasize the ways in which donors remain present in—rather than absent from—their families, albeit often symbolically (Graham, 2014), and that single mothers may be especially likely to discuss the donor with their children as part of a ‘family reverie’ (Ehrensaft, 2007) in which there exists a ‘fantasy father’ (Ehrensaft, 2000; Hertz, 2006). However, researchers have also called into question the apparent uniformity of these user groups—delineated according to relationship status—with regard to their thoughts and feelings about the donor. In one study, single women were found to be likely to differ in their thoughts about the donor according to whether they had used anonymous or known donation (Hertz and Ferguson, 1997; Hertz, 2006). In another, approximately half of all single mothers who had conceived using an anonymous donor described a desire to know more about him (Landau and Weissenberg, 2010). A third study found no differences between single and coupled recipients with regard to preferences for identity-release or anonymous donation (Scheib *et al.*, 2000), and a fourth identified that both single and coupled recipients of donor sperm viewed the donor's health, name and access to a photograph as highly important (Rodino *et al.*, 2011a). This research thus points to the existence of diversity within different user groups regarding thoughts and feelings about the donor, and the significance attributed to both donor type and donor information.

By way of explaining these findings, it has been suggested that interest in donor information may be guided by recipients' intentions to tell their children about their conception (Scheib *et al.*, 2003). However, while studies have also indicated that single women may be more likely than their heterosexual couple counterparts to have shared, or plan to share, this information with their child (Leiblum *et al.*, 1995; Klock *et al.*, 1996; Scheib *et al.*, 2003), the impact of identity-release donation upon the donor narratives of single mothers is not yet clear. Indeed, given the discrepant findings with regard this user group, understanding the factors that may determine the ways in which single mothers think and feel about the sperm donor is now crucial.

This study thus seeks to provide greater insight into the ways in which single mothers by donor insemination conceptualize the donors involved in their child's conception by focussing on the narratives produced about the donor. It explores the factors that impact upon mothers' representations of the donor, and specifically elucidates the impact of legislation upon thoughts and feelings about him.

## Materials and Methods

### Sample characteristics

Participants were recruited through the London Women's Clinic, the largest fertility clinic in the UK that offers donor insemination to single women. Mothers had at least one child aged between 4 and 9 years conceived by sperm donation, were heterosexual and were currently without a live-in and/or long-term partner. These inclusion criteria were determined by mothers' involvement in a larger study of family functioning and child development in single-mother families (Golombok *et al.*, unpublished data).

Of a total of 63 women contacted by the research team, 46 agreed to take part, giving a response rate of 73%. Mothers' ages ranged from 32 to 51 years (mean = 44.17, SD = 4.00). The majority had one child (*n* = 30, 65%) and had never been married (*n* = 39, 85%). Twenty mothers (43%) had used an anonymous donor and 26 (57%) had used an identity-release donor. The mean age of children conceived using an anonymous donor was 6.9 years (SD = 1.30). The mean age of children conceived using an identity-release donor was 4.5 years (SD = 0.76).

### Interviews and analysis

Each participant took part in a qualitative interview conducted by one of three researchers. The interview schedule was developed according to researchers' interest in the meanings single mothers attribute to their experiences, and the representations they form of family life (Zadeh *et al.*, 2013). To ensure question efficacy and cross-interviewer consistency, pilot interviews were conducted by each researcher in the presence of a second researcher.

All interviews were conducted in participants' homes and lasted ∼90 min, with the shortest being 37 min, and the longest ∼3 h. They were all audio-recorded. Participants were asked about their experience of choosing a donor, their thoughts and feelings about the donor pre- and post-birth, the information they were given, currently have, and would like about the donor, and their discussions with other people, including their children, about him.

Data were analysed using Atlas.ti according to the principles of thematic analysis (Braun and Clarke, 2006). Important to the first stage of data familiarization, all interviews were transcribed verbatim by the primary interviewer (S.Z.). Identifying information was removed from all transcripts, and pseudonyms were used to ensure participants' anonymity. A total of 112 codes across interview scripts were generated. Codes specifically relating to mothers' discussions of the sperm donor (of which there were 38 in total) were then subject to separate thematic analyses and refined to produce two main themes and six subthemes reflective of the dataset as a whole. Regular peer debriefing and the completion of a systematic data audit served to ensure the validity of results (Flick, 2014).

Ethical approval for the study was given by the University of Cambridge Psychology Research Ethics Committee. Written informed consent was obtained from all participants.

## Results

Findings indicated that participants' thoughts and feelings about the donor could be understood in relation to two overarching themes of ‘presence’ and ‘absence’. Most (*n* = 27) mothers talked about the donor as an ‘absent presence’ who was in one or more ways symbolically significant to family life, and were likely to describe the donor as (i) having given them a gift (the ‘gift giver’), (ii) having provided their child with genetic material (the ‘gene-giver’), and (iii) as in some sense part of ‘traditional’ family life (the ‘potential partner’). Others (*n* = 16) talked about the donor as an ‘absence’ and described him as (i) a figure about whom little or nothing was known (the ‘unknown’), (ii) donor sperm, rather than a sperm donor (‘part of a process'), and a person whose contribution was intangible (‘out of sight, out of mind’). Within this second subgroup, mothers expressed either positive or negative feelings about the donor's ‘absence’.

The themes of ‘presence’ and ‘absence’ were found to characterize participants' narratives both in terms of how they specifically described the donor, and in terms of the strategies they described as facilitating their representation of the donor as either a ‘presence’ or an ‘absence’. A minority (*n* = 3) of mothers appeared to currently represent the donor as *both* an ‘absent presence’ and an ‘absence’ in equal measure, often vacillating between the two in the course of the interview.

In the ‘present’ group, 9 mothers had used an anonymous donor, and 18 mothers an identity-release donor. In the ‘absent’ group, 10 mothers had used an anonymous donor, and 6 had used an identity-release donor. Two of the three mothers who described the donor as both ‘present’ and ‘absent’ had used an identity-release donor. The third had used an anonymous donor. These mothers were later categorized as being in both groups for the purpose of illustrating results.

## The donor as an ‘absent presence’

### The gift-giver

The majority of mothers described the donor as having given them a ‘gift’, for which their gratitude was unquantifiable. Some mothers suggested that they had specifically chosen a donor on the basis that he too saw his donation in such terms:

In our donor's particular case, we know that he had an altruistic reason for doing it … He'd observed infertility in his family, and had agreed to help another woman, which is, er, much better than it being a … student jerking into a cup to pay to go out to buy a few more beers.

(Anna, anonymous donation)

Other mothers explained that although they were not given information from the clinic about the donor's reason for donating, they were assured that the donor was a ‘kind man’, since he had chosen to donate his sperm in the first place. However, although several mothers stated that they would like to meet the donor to personally express their gratitude, others appeared to conceptualize the donor's gift as marking the end of a specific—albeit extremely generous—exchange:

I only think of him as a very kind and generous soul. That's almost the deal. He's done his bit … done and dusted.

(Barbara, anonymous donation)

Responding to questions about the donor's possible future involvement in their families, some mothers expressed a belief that donors who specifically made a donation after 2005 would, as ‘kind men’, be willing to have contact with donor-conceived children:

What's the point of knowing that you're gonna get knocks on your door in 18 years' time, to say ‘I don't want to know you?’ I know these men, who are becoming the donor dads, are gonna be happy to have someone who's related to them come and say hello to them.

(Penny, identity-release donation)

Many mothers who had begun the process of telling their children about their donor conception explained that they had described the donor as a ‘kind man’, although some expressed some discomfort about doing so. This was the case irrespective of whether mothers had used an anonymous or identity-release donor:

I have to be careful that I don't embellish him with stuff that I don't know he has.

(Anna, anonymous donation)

We don't really go into—we don't build up a fantasy. We just [talk about] some man who was really kind, and allowed mummy to use the special stuff.

(Mog, identity-release donation)

Other mothers indicated that although they had described the donor to their child as a ‘kind man’ who had given them a gift, they took great care to describe him in terms that distinguished him from a father figure. Several mothers, for example, made a distinction between a ‘daddy’ and a ‘father’; others clarified that the ‘donor daddy’ was not a ‘day-to-day daddy’ or a ‘normal dad’.

### The gene-giver

Alongside describing the donor as ‘gift-giver’, the majority of mothers explicitly designated the donor as ‘present’ in their family narratives as part of the story of where their child ‘came from’. For most, the donor was seen to be the carrier of specific genetic traits, and as somebody who had therefore provided their child with some of their genetic material. Some mothers described having chosen their specific donor on the basis of this belief:

I wasn't bothered about IQ in the slightest. I'm sure that's more social than genes. I was bothered about character, and … very vainly, what he looked like.

(Emma, identity-release donation)

Being not terribly tall myself, I just thought that if I had a boy then … at least I wanted to give him some extra genes for height.

(Juliet, anonymous donation)

At the same time, several mothers reflected upon how their thoughts and feelings about the donor had changed post-birth, and especially after acknowledging that their children possessed particular characteristics and mannerisms that they could not identify in themselves:

I'm really intrigued to know what he's like … whether there's similarities and whether there are things about her that I'm not like … so that must come from someone else … There must be mannerisms and stuff that's similar.

(Gilly, identity-release donation)

One mother specifically suggested that the identification of these characteristics in her child made the donor continually ‘present’ for her:

Sometimes when I look in [my child's] eyes, cos his eyes aren't anything like any of our family, that's the link I feel with the dad, his eyes.

(Holly, anonymous donation)

Alongside envisioning the donor as a ‘giver’ of genes, several mothers specifically suggested that the donor's genetic contribution meant that he represented the ‘root’ of their child's identity, often likening the experience of donor-conceived children to adopted children who search for information about their origins. A minority of mothers stressed that this was not how they had thought about the donor during treatment:

Now I realise for them how important it will be … because it's part of them.

(Carol, identity-release donation)

I think before … I would have thought it would be better to be anonymous, but then I thought oh well it doesn't matter, and then when I thought about it, I think it's really important because … if she ever wants to know, which, you know, she may do, I think that's a really key part for her.

(Tanya, identity-release donation)

Some mothers also remarked that further information from the HFEA had been especially helpful, not only in allowing them to think about the donor in greater detail, but also in facilitating their knowledge of their child's ‘genetic background’ in general. Several mentioned the significance of information provided about ‘donor siblings'—other children who had also been created through the use of their donor's sperm. For some participants, however, knowing more about these children served to provoke a great deal of anxiety:

That was my overriding focus, the best way and the quickest way of getting a baby … It's awful isn't it? Like, worst, I spend more time choosing, I don't know, things in the supermarket, isn't that strange? Or a pair of shoes or something. Now of course I'm thinking … there's all these half brothers and sisters out there in the world, which could cause issues as they get older.

(Barbara, anonymous donation)

I feel very protective … We have our own family, and I want to protect them. The whole sibling [thing] … I could go on and contact all these people, but I have no control about them.

(Carol, identity-release donation)

### The potential partner

Several mothers explained that the donor was a figure who had ‘presented’ himself as a ‘potential partner’, stating that they had chosen a donor on the basis of characteristics that they were attracted to:

I think it was important to me that they were educated, just because that reflects my own kind of beliefs and what I would look for in a partner, I guess, so that's the only way I could judge it really. And yeah, someone tall, because I like tall men.

(Sandy, identity-release donation)

In so doing, mothers described how fertility clinic staff had been instructive in their decision-making and had provided them with additional information about the donor:

I remember saying to [the nurse], if you had to date one of them, who would you go out with? And we had a very funny conversation around that … I mean every now and then she'll say a little bit, she'll put a little meat on the bones, nothing much, but that makes the person come alive a bit.

(Susan, identity-release donation)

Although it was rare for mothers to discuss the donor as a potential future partner, one mother did describe wondering whether ‘there might even be a spark’ (Melanie, anonymous donation), referring to the romantic potential of future contact with the donor. Some also suggested that it was regrettable that the donor was ‘missing out on this wonderful thing’ (Gilly, identity-release donation) he had helped to create, thereby alluding to ‘traditional’ family circumstances.

## The donor as an ‘absence’

### The unknown

Several mothers commented upon the donor as an ‘unknown’ entity; some responded to questions about the information they had about the donor in ways that clearly indicated that they had not incorporated information about him into their family narratives, but had rather rendered him ‘absent’:

His age, no I don't think his age was on it, no. Um, what else was on it, oh I can't remember now! No, there were some other things, but I can't remember what they were.

(Zoe, identity-release donation)

I was [given information] but I never wrote any of it down, and I never thought to … ask them to write it down … After so many tries, they did so many different donors and I think I'm probably getting muddled up.

(Abby, anonymous donation)

Mothers describing the donor as ‘unknown’ varied in their thoughts and feelings about their lack of information about him. Although it is possible that the participants quoted above were in fact not comfortable to share information about the donor with researchers, others explicitly stated that they had refused information:

I don't really know much about the donor … No I don't know anything. I know they, they offered to give me a [pen] sketch, and I said no.

(Rosa, identity-release donation)

One mother indicated that in order to maintain her representation of the donor as ‘unknown’ she had chosen not to have more children after legislative changes to donor anonymity:

I didn't know if I had another child that the reasons could be disclosed, or there could be some claim or something, just something that would come back and haunt me in the future.

(Kathy, anonymous donation)

Relatedly, although several mothers expressed indifference about the fact that they could not contact the donor, others reflected upon the practice of anonymous donation—and their resultant representation of the donor as ‘unknown’—in highly negative terms. This was particularly true of mothers who wanted more information about their identity-release donor, but were currently unable to access it, and for those who at the time of treatment had believed their anonymous donor to be identity-release:

I thought he was a known donor, and he actually isn't … The donor probably gives me the creeps, actually. He's this strange person that she will never, you know … a white blank page.

(Cynthia, anonymous donation)

Some mothers who had received further information about their identity-release donor nevertheless also described him as an ‘absence’ who was difficult to think of as anything other than a ‘ghost’ or ‘stranger’:

It doesn't matter how much stuff you read about someone on a piece of paper, you can never know what they're like, really, unless you meet them, and you're not gonna meet them.

(Jackie, identity-release donation)

Mothers describing the donor as ‘unknown’ also differed in their thoughts and feelings about how this might impact upon the future conversations they may have with their children about him. While some mothers expressed some anxiety around this issue, others described how they hoped that their children would follow suit in ‘absenting’ the donor from their families in the future:

I think maybe as she gets older it will get a bit more difficult because, you know, she'll want to ask more questions and want more answers and it may get to the stage where I can't answer some of her questions … about her donor …

(Lesley, anonymous donation)

I'm hoping it won't be such a big deal that she'll go, ‘I can't really be bothered [to contact him]’.

(Emma, identity-release donation)

### Part of a process

Mothers who mostly described the donor as an ‘absence’ were also likely to stress that they had used a fertility clinic to create their family, and to emphasize the use of ‘donor sperm’—rather than a ‘sperm donor’—in their path to parenthood:

The clinic is under an obligation to make sure that there were no [problems in the] medical history involved, so I'm quite happy that they did their job properly.

(Imogen, anonymous donation)

The sperm's tested so much they, like any defect or anything just doesn't get through.

(Jackie, identity-release donation)

Some mothers specifically described how they had chosen to undergo donor insemination in a clinical setting because of the potential risks involved in using a known donor or a donor outside of a licensed fertility clinic who may later become ‘present’ in their child's life. Those who conceptualized the donor in this way also tended to describe him to their children as ‘part of a process' that led to them becoming a mother, instead emphasizing the contribution others had made to their child's conception, such as fertility clinic staff:

So I just told her that um, a nurse … identifying a person, rather than just a strange, um, helped mummy to have you in a very very special way.

(Cynthia, anonymous donation)

### Out of sight, out of mind

The clinic was additionally described as significant by participants who labelled the donor as an ‘absent’ figure who was also ‘out of sight’. Several mothers explained that they were advised by clinic staff to use a donor with similar physical attributes to themselves, so that there would be ‘no obvious signs of someone else being involved’ (Lorna, identity-release donation). Indeed, mothers mostly described choosing a donor based on the fact that his characteristics aligned with their own, or those of their family members.

Although some of the mothers who chose to use a physically similar donor described being able to identify his characteristics in their children, others suggested that they had been successful in physically—and therefore genetically—‘absenting’ the donor from their families:

I didn't want [my child] to look different to us. I mean it probably doesn't matter anyway, because at the end of the day he's my dad's double, isn't he? … I don't think any other genes in that sense got a look in.

(Victoria, anonymous donation)

As it happens, my daughter's the spitting image of me as a child … and [my son] is very like the men in our family … so it's obvious my genes were the strongest anyway!

(Imogen, anonymous donation)

In addition to mothers suggesting that the donor's ‘absence’ was reinforced by him being ‘out of sight’, some participants explained that the donor was not relevant to their everyday experience of family life. Portraying the donor as ‘out of mind’, these mothers described an overarching lack of interest, and a general lack of wondering about him or his life:

I don't really talk about him as a person, cos he's not really a person, is he, in our lives? He's not, we don't see him on a personal basis.

(Emma, identity-release donation)

I forget that there is a donor … it's not a big part of our lives.

(Joanne, identity-release donation)

## Discussion

The findings of this study reveal marked diversity in single mothers' representations of the donors involved in their paths to parenthood. While most mothers conceptualized the donor as an ‘absent presence’, that is, as a figure important to—although not physically present in—their families, others were more likely to describe the donor as an ‘absence’. The analysis revealed that mothers in the latter group differed in their feelings about the donor's ‘absence’, such that for some, the perceived inability to make the donor ‘present’ was a source of difficulty and anxiety, and for others, it was not. These findings incite reflection upon the existing literature on representations of the donor in single-mother families. In contrast to the idea that single women may be congruent in their views of the donor (Ehrensaft, 2000; Graham, 2014), the present findings are in keeping with research that has painted a more nuanced picture of this particular user group (Landau and Weissenberg, 2010).

Indeed, the experiences of the participants in this study show that complex and changeable feelings about the donor may be experienced by mothers in both one and two-parent families. As in previous research that has indicated that mothers' feelings about the donor are likely to change over time, and especially post-birth (Vanfraussen *et al.*, 2001; Kirkman, 2004a; Hertz, 2006; Ehrensaft, 2007; Ehrensaft, 2008; Grace *et al.*, 2008; Burr, 2009; Landau and Weissenberg, 2010; Indekeu *et al.*, 2014; Wyverkens *et al.*, 2014), participants often reported feeling rather differently about the donor once having had a child. For some mothers, observing their children's physical and emotional development had resulted in a heightened awareness of the existence of the donor, particularly once having identified characteristics in their children that they perceived to be unlike their own. For others, although significant during the treatment process, the donor had since become irrelevant to their day-to-day experience of family life. Moreover, receiving little information about the donor during the process of treatment was retrospectively thought to be regrettable by some, whereas for others, specific information, including information about donor siblings, was now thought of as a potential intrusion into family life. That different treatment phases (Stuart-Smith *et al.*, 2012) and transitions from pregnancy to parenthood (Indekeu *et al.*, 2014) may be marked by very different thoughts and feelings about the donor is an important finding for practitioners charged with counselling recipients of donor insemination at different stages of the process. In fact, the findings of this study seem to suggest that despite HFEA guidelines, current counselling with regard to the type of information that is or may become available about the donor and other children conceived using his gametes may be insufficient.

The findings also indicate that despite changes to legislation in 2005, single recipients of donor sperm do not necessarily desire to use an identity-release donor, either at the time of treatment, or indeed at all. Moreover, it is worthy of attention that some of the participants who had used treatment after 2005—that is, with an identity-release donor—had no interest in receiving information about the donor, and no desire for him to be involved in future family life. In spite of the suggestion that sociolegal shifts towards identity-release donation have created difficulties specific to parents who seek to minimize the contribution of the donor in their family narratives (Grace *et al.*, 2008), findings therefore suggest that this may not be true of all recipients of donor sperm in identity-release programmes. Given that the majority of the mothers in this study also reported having shared, or having an intention to share, donor conception information with their children (Freeman *et al.*, unpublished data), the analysis also complicates the idea that donor information may be especially desired by recipients who intend to tell their children about their donor conception (Scheib *et al.*, 2003). Although findings seem to suggest that current HFEA guidelines about counselling on the topic of telling children about their conception are being put into practice, it may be the case that dissemination of information about the varying needs of donor-conceived individuals in adolescence and adulthood (Freeman, 2015) is currently lacking.

It is worth highlighting that in this study, participants with anonymous donors had children who were on average ∼2 years older than the children whose mothers had used identity-release donors. As it has been shown that understandings of donor conception increase incrementally during childhood (Blake *et al.*, 2014), it might be expected that children conceived with anonymous donors would be asking more questions about the donor than their younger counterparts conceived within identity-release programmes. As such, the finding that mothers in both donor programmes identified him as either a ‘presence’ or an ‘absence’ may indicate something about the conversations about the donor (or indeed, lack of them) that are currently taking place between mothers and children in single-mother families. Unlike previous findings that mothers and children may be involved in co-constructing an image of the donor (Hertz, 2006), the findings of this study in fact suggest that like other groups seeking treatment (Mamo, 2005), single women may rather be influenced in their thoughts and feelings about the donor by conversations with fertility clinic staff. It is possible that these informal practices vary from clinic to clinic. It is moreover notable that several websites offering donor sperm now include information about ‘staff impressions' of the donor, suggesting that this process of representational co-construction has also become more formalized in recent years.

Amongst the majority of mothers who did discuss the donor as a symbolic ‘presence’, the donor's reasons for donating were highlighted as important. This finding has been echoed in research on single women seeking fertility treatment (Graham, 2014), and in research on lesbian and heterosexual couples using donor insemination (Chabot and Ames, 2004; Mamo, 2005), with a recent study finding that although not opposed to financial compensation, recipients of donor sperm may nevertheless prefer their donor to have altruistic motivations (Ravelingien *et al.*, 2015). The reason for donation was also described by some participants as allowing an insight into the donor's feelings about future contact, a factor previously shown to be important to single women making decisions about donor insemination (Rodino *et al.*, 2011b). Although current recommendations in the UK are such that clinicians are advised to encourage donors to provide as much information as possible, the fact that reasons for donation were deemed significant by some recipients seems to indicate that amendments to existing HFEA guidelines—specifically with regards to this information—may now be warranted.

Interestingly, the results of this study illustrate that regardless of whether mothers represent the donor as a ‘presence’ or ‘absence’ in their family narratives, they do so by drawing upon a specific discourse of genetic heritability that either emphasizes the genetic connections between donor and child as significant, or discounts the contribution of the donor as genetically insignificant. Given that the time at which this study was conducted has been described as the ‘age of genetic essentialism’ (Freeman, 2014), this emphasis on genetic connections is perhaps unsurprising. These findings do, however, contrast with previous research that has indicated that parents seeking to discount the donor's contribution are likely to emphasize the power of social, rather than genetic, ties (Kirkman, 2004a). As Hudson and Culley (2014) have advised practitioners working with minority ethnic communities seeking fertility treatment, it is recommended that fertility clinic staff guard against making assumptions about single recipients' thoughts and feelings about genetic connections, as well as their ideas about what constitutes genetic information, and what does not.

This study provides a rich insight into the donor representations of single mothers with donor-conceived children. The qualitative nature of the research has enabled an in-depth investigation of mothers' meaning-making about the donor and sheds light on the thoughts, feelings and experiences of women who have children in this way. The findings from qualitative research studies such as this may not be and ought to be considered with caution. However, it appears that single mothers with donor-conceived children have diverse views and experiences regarding the donor that are not necessarily determined by donor type. Their representations may also be subject to change over time. As discussed, these findings have significant implications for both the counselling and treatment of single women seeking fertility treatment, especially in contexts in which only one type of donor (anonymous or identity-release), and/or specific donor information, is made available. The study makes clear that marital status cannot be assumed to determine recipients' thoughts and feelings about the donor or donor information. Nor can these thoughts and feelings be assumed to be static. Given that the children in the families studied were still of a relatively young age, longitudinal research on single-mother families formed through donor insemination is now recommended.

## Authors' roles

All authors were involved in study design, data acquisition, analysis and interpretation. This manuscript was drafted by S.Z. and has been approved by all authors.

## Funding

This study was supported by the Wellcome Trust [097857/Z/11/Z]. Funding to pay the Open Access publication charges for this article was provided by the University of Cambridge.

## Conflict of interest

The authors have no conflicts of interest to declare.
